# Optimization of Magnetic Cobalt Ferrite Nanoparticles for Magnetic Heating Applications in Biomedical Technology

**DOI:** 10.3390/nano13101673

**Published:** 2023-05-18

**Authors:** Diana Zahn, Joachim Landers, Marco Diegel, Soma Salamon, Andreas Stihl, Felix H. Schacher, Heiko Wende, Jan Dellith, Silvio Dutz

**Affiliations:** 1 Institute of Biomedical Engineering and Informatics (BMTI), Technische Universität Ilmenau, D-98693 Ilmenau, Germany; 2 Faculty of Physics and Center for Nanointegration Duisburg-Essen (CENIDE), University of Duisburg-Essen, D-47057 Duisburg, Germany; 3 Leibniz Institute of Photonic Technology (IPHT), D-07745 Jena, Germany; 4 Institute for Organic Chemistry and Macromolecular Chemistry, Friedrich-Schiller-University Jena, D-07743 Jena, Germany; 5 Jena Center for Soft Matter (JSCM), Friedrich-Schiller-University Jena, D-07745 Jena, Germany; 6 Leupold Institute for Applied Natural Sciences (LIAN), Westsächsische Hochschule Zwickau, D-08056 Zwickau, Germany

**Keywords:** magnetic nanoparticles, cobalt ferrite, magnetic heating

## Abstract

Using magnetic nanoparticles for extracorporeal magnetic heating applications in bio-medical technology allows higher external field amplitudes and thereby the utilization of particles with higher coercivities (H_C_). In this study, we report the synthesis and characterization of high coercivity cobalt ferrite nanoparticles following a wet co-precipitation method. Particles are characterized with magnetometry, X-ray diffraction, Mössbauer spectroscopy, transmission electron microscopy (TEM) and calorimetric measurements for the determination of their specific absorption rate (*SAR*). In the first series, Co_x_Fe_3−x_O_4_ particles were synthesized with x = 1 and a structured variation of synthesis conditions, including those of the used atmosphere (O_2_ or N_2_). In the second series, particles with x = 0 to 1 were synthesized to study the influence of the cobalt fraction on the resulting magnetic and structural properties. Crystallite sizes of the resulting particles ranged between 10 and 18 nm, while maximum coercivities at room temperatures of 60 kA/m for synthesis with O_2_ and 37 kA/m for N_2_ were reached. Magnetization values at room temperature and 2 T (M_RT,2T_) up to 60 Am^2^/kg under N_2_ for x = 1 can be achieved. Synthesis parameters that lead to the formation of an additional phase when they exceed specific thresholds have been identified. Based on XRD findings, the direct correlation between high-field magnetization, the fraction of this antiferromagnetic byphase and the estimated transition temperature of this byphase, extracted from the Mössbauer spectroscopy series, we were able to attribute this contribution to akageneite. When varying the cobalt fraction x, a non-monotonous correlation of H_C_ and x was found, with a linear increase of H_C_ up to x = 0.8 and a decrease for x > 0.8, while magnetometry and in-field Mössbauer experiments demonstrated a moderate degree of spin canting for all x, yielding high magnetization. *SAR* values up to 480 W/g (@290 kHz, 69 mT) were measured for immobilized particles with x = 0.3, whit the external field amplitude being the limiting factor due to the high coercivities of our particles.

## 1. Introduction

Magnetic nanoparticles (MNP) have been extensively studied for medical applications, including therapeutic hyperthermia [1,2,3]; drug targeting where MNP are used as vehicles for the transport of therapeutic agents [4,5]; or imaging that utilizes MNPs as contrast agents [6,7]. Each of the approaches mentioned above requires the particles to be applied intracorporeally, leading to restrictions for the particles concerning their biocompatibility, but also for the external magnetic fields used to navigate or heat the particles. Safety restrictions limit an external alternating magnetic field to H×f = 5·10^9^ Am^−1^s^−1^ [8], to prevent unspecific heating of tissue by eddy currents and to ensure patients’ comfort. 

In addition to these intracorporal medical approaches, MNP are also promising candidates for medically related extracorporeal approaches: their ability to be moved by a magnetic gradient can be used to extract pathogens from specimens and use them as magnetic markers for immunomagnetic separation [9,10]. Furthermore, their mechanism of generating heat can be used extracorporeally, for example as thermal markers in place of the common colorimetric markers used in immunoassays. In this context, external fields with remarkably higher field amplitudes can be used to heat the particles, enabling the use of hard magnetic MNP with higher anisotropy. Such particles are not of interest for intracorporal heating applications, since the required field amplitude to efficiently heat these particles needs to be two or three times the particles’ coercivity [11], exceeding the limit for patient safety. 

Commonly used iron oxide nanoparticles can be tuned towards a higher anisotropy by changing their shape from spherical to cubic, disc-like or elongated needles [12,13,14,15]. Increasing their size while remaining in a single domain state also increases their coercivity [16,17,18]. Another approach to the synthesis of particles with large coercivities is the introduction of metal atoms such as Zn, Mg, Ba or Co into the crystal lattice to synthesize ferrites. Starting with the inverse spinel magnetite (Fe_3_O_4_ or [Fe^3+^]_tetra_[Fe^2+^Fe^3+^]_octa_O_4_), where Fe^3+^ ions are distributed equally between octahedral and tetrahedral sites and Fe^2+^ ions are located on the octahedral site [19], Co^2+^ ions can replace the Fe^2+^ ions. Particles of pure cobalt ferrite, where Fe^2+^ is completely replaced by Co^2+^, are well known to exhibit exceptionally high coercivities of up to several hundred kA/m at room temperature [20,21]. As stated above, an external magnetic field used to heat such particles would need an impractically high field amplitude. Therefore, a partial replacement of Fe^2+^ by Co^2+^ can be beneficial for obtaining materials with the formula Co_x_Fe_3−x_O_4_, resulting in coercivities and magnetization values between magnetite and cobalt ferrite. 

When examining the literature that focuses on this kind of partially substituted magnetite particles, controversial results can be found regarding the correlation between cobalt content x and resulting particle properties. The coercivity of H_C_, for example, is found to increase monotonously with cobalt content [22] or to reach a maximum and then decrease for higher cobalt contents towards pure cobalt ferrite (x = 1) [23,24]. In addition, no clear trend for the saturation magnetization (M_S_) can be found—M_S_ can increase with more cobalt [25], decrease [22,26], or show a maximum for intermediate cobalt contents [24,27]. Crystal size and lattice parameters show linear trends with rising cobalt content, but whether this is an increasing or decreasing trend is not consistent, and most likely depends on the degree of oxidation from magnetite to maghemite for the particles with no or small amounts of cobalt [22,23,27,28]. 

To develop efficient cobalt-substituted magnetite particles that match the application specific requirements by tuning the particle properties, the above-mentioned correlations need to be understood. Therefore, in the first series of experiments we used a co-precipitation method to synthesize a broad range of cobalt ferrite particles (x = 1) with varied synthesis parameters, to evaluate the influence of reaction temperatures, durations, and atmosphere (O_2_ or N_2_) on the resulting particles. In the second series, we used fixed optimized synthesis parameters to produce partially substituted magnetite particles of Co_x_Fe_3−x_O_4_ with x = 0, 0.3, 0.5, 0.7, 0.8, 0.9, 0.95 and 1, to study the correlations between cobalt content x and the resulting particle properties. The particles were characterized using vibrating sample magnetometry (VSM), X-ray diffraction, transmission electron microscopy, Mössbauer spectroscopy as well as calorimetric measurements for determination of the heating capability. 

As the main conclusions, we found thresholds for the reaction temperatures, which, if exceeded, would lead to the formation of crystalline impurity phases—apart from CoFe_2_O_4_—and the significant decrease of magnetization. Oxygen-free synthesis under N_2_ atmosphere leads to higher magnetization values and decreasing H_C_ compared with synthesis under air. With increasing amounts of cobalt, H_C_ at 300 K reaches a maximum at x = 0.8, while M_RT,2T_ ranges from 71 to 77 Am^2^/kg for 0 < x < 0.9 and slightly decreases. In-field Mössbauer experiments were employed to analyze the particles‘ magnetic structures, check for the presence of Fe^2+^, determine the Co and Fe ion site occupation and its influence on the particles’ magnetization, and to evaluate the correlation between Co content x and the samples’ degree of spin frustration. 

## 2. Materials and Methods

### 2.1. Synthesis of Cobalt Ferrite Nanoparticles

Cobalt ferrite nanoparticles were prepared using a wet co-precipitation method. For this method, iron(III) chloride hexahydrate and cobalt(II) chloride tetrahydrate are dissolved in 120 mL deionized water in a two-neck glass flask under stirring in a water bath, heating the salt solution to the addition temperature (T_add_). An amount of 12 mL of a 3M sodium hydroxide solution is added with a syringe pump over a specified duration, hereafter called duration of NaOH addition or d_add_. The flask is then transferred to an oil bath, heating the solution to a higher temperature named end temperature or T_end_ and kept in this bath under stirring for a defined time, named duration of reaction or d_react_. For a structured analysis of the influence of the named synthesis parameters on the resulting particle characteristics, for the first part of this study (hereafter named series 1) we varied the parameters over a broad range. Each set of parameters was used for the synthesis of three particle batches to enable a basic statistical analysis of the results. Additionally, all parameter sets were reproduced under the exclusion of oxygen by washing the used solutions with nitrogen prior to the synthesis and keeping the flask under nitrogen atmosphere throughout the whole reaction. Table 1 shows the used settings for the four parameters T_add_, d_add_, T_end_, d_react_. When one of the parameters is varied, the remaining parameters are kept constant at the value written in bold in the table. For the parameter “duration of addition”, 0 min means an addition of the total volume of NaOH solution as a single bolus over a period of seconds. For evaluation of the synthesis parameters, a stochiometric cobalt-to-iron ratio was used in this series of particles to synthesize completely substituted cobalt ferrite nanoparticles with x = 1 and a ratio of Co^2+^ ions to Fe^3+^ ions of 1:2. When needed, samples are named in the following results as O_2_/N_2__d_add__T_add__T_end__d_react_.

As a second part of the study (hereafter named series 2), the amount of cobalt was altered and iron(II) chloride tetrahydrate was introduced to the synthesis. By varying the ratio of Co^2+^ ions to Fe^2+^ ions and while keeping the amount of Fe^3+^ ions constant, a series of Co_x_Fe_3−x_O_4_ particles with varying cobalt content was synthesized. The percentage of Fe^2+^ ions replaced by Co^2+^ ions is named cobalt fraction x in the following and was evaluated in the range from 0 (no Co^2+^ ions) to 1 (Fe^2+^ is replaced completely by Co^2+^) via the intermediate steps x = 0.3, 0.5, 0.7, 0.8, 0.9, 0.95. Three batches of particles were synthesized for each x. In those experiments, the synthesis parameters were set to T_add_ = 40 °C, d_add_ = 2 min, T_end_ = 97 °C and d_react_ = 90 min and the synthesis was carried out under nitrogen atmosphere, to minimize nanoparticle oxidation. These values were chosen considering the results of series 1 of the study, since they enable a high H_C_ while providing a high M_RT,2T_ at the same time.

After synthesis, particles were washed magnetically with deionized water and stored in deionized water. About 200 mg of each sample were dried under air at room temperature to produce powders for following characterization measurements.

### 2.2. Characterization of Cobalt Ferrite Nanoparticles

#### 2.2.1. Transmission Electron Microscopy (TEM)

For the evaluation of shape and size of the resulting particles, TEM images were acquired for the samples of series 2. One sample for each cobalt substitution rate x was used as a dry powder and suspended in micropure water at a concentration of 5 mg/mL. The sample was deposited on a carbon support film on a 400-mesh copper grid manufactured by Quantifoil Micro Tools (Großlöbichau, Germany). The films were hydrophilized in an Argon plasma produced by a Diener Electronics (Ebhausen, Germany) plasma oven for 120 s prior to sample deposition. An amount of 10 μL of the suspension was then placed on the film, the excess blotted off using filter paper, and allowed to air dry. TEM images were acquired with a 200 kV FEI Tecnai G^2^ 20 (Hillboro, OR, USA) using a 1 k × 1 k Olympus MegaView camera (Münster, Gemany) with the acceleration voltage set to 120 kV.

#### 2.2.2. Magnetometry

For a comparative evaluation of the magnetic parameters of the particle batches synthesized in series 1 of the study, VSM measurements at room temperature were conducted using an MSE-EZ9, Microsense, Lowell, MA, USA. A weighed amount of the particle powders, around 3 to 5 mg, was transferred to 3D-printed polymeric measurement vials and hysteresis curves were recorded within a field amplitude of ±2 T, using a sampling interval of 0.2 T for the saturated branches of the curve and 0.005 T between ±0.1 T to determine coercivity more precisely. Magnetization at room temperature and a field of 2 T (M_RT,2T_) as well as coercivity (H_C_) were extracted from each measurement and the mean values were calculated for the three particle batches synthesized with the same parameter set.

For a more detailed evaluation of the magnetic behavior of the particles synthesized in series 2 with varied cobalt substitution ratios, magnetometry measurements at 5 and 300 K up to a field of ±9 T were carried out using the VSM option of a Quantum Design PPMS DynaCool (San Diego, CA, USA) for one representative sample out of each set of three particle batches with the same x. 

#### 2.2.3. Mössbauer Spectroscopy

Mössbauer spectroscopy was utilized for selected samples of series 1 and all samples of series 2, where again one representative sample for each x was characterized. Mössbauer spectra were measured in transmission geometry using a constant acceleration driving unit and ca. 20 mg/cm^2^ of oxide nanoparticle powder. To study sample composition based on magnetic phase transitions, spectra between 4 and 300 K were recorded in a closed-cycle cryostat (SHI-850-5, Lake Shore Cryotronics, Westerville, OH, USA), while in-field spectroscopy was performed using a l-He bath cryostat with a superconducting split-coil magnet allowing maximum magnetic fields of 10 T (Spectromag-4000-10, Oxford Instruments, Abingdon, UK) to analyze magnetic alignment behavior in more detail. Upper estimations of the hydroxide fraction are based on the relative spectral intensity of the corresponding subspectrum observed in spectra at 300 K.

#### 2.2.4. X-Ray Diffraction (XRD)

Crystal structure and size of the particles were evaluated using X-ray diffraction. Diffractograms were measured using a Panalytical X’Pert Pro MPD theta-theta diffractometer (Malvern Panalytical Ltd., Malvern, UK) with parallel beam setup and 1D line detector in scanning mode. For fluorescence reduction, the lower discrimination threshold of the PIXCEL detector was set to 45.5%. The divergence and anti-scatter slits were 0.5° and beam mask was 10 mm. Measurements were taken from 15° to 85° 2θ with 2 h measurement time. Phase identification and crystallite sizing was undertaken with Malvern Panalytical Highscore Plus V. 4.9 [29] and the databases ICDD pdf2 from 2001 and COD 2021. Crystallite sizing was completed with the “size-strain-analysis-R”-method (SSA-R) for the phase peaks, which provides more accurate values compared with the commonly used Scherrer method, which only takes one peak into account. Previously, the instrumental broadening was determined for the X-ray optics used with a 100% anatase powder sample (TiO_2_). The powder samples were ground by hand with a mortar prior to measurement. The sample holder was a “zero background holder” (ZBH) with a recess of 15 mm diameter and 0.2 mm depth, into which the powder was filled flush. The ZBH rim was adjusted to the target height of 1 mm (± 1 µm) to avoid peak shift due to height error. From the highest peak ((311) for CoFe_2_O_4_) the interplanar spacing d was used to calculate the lattice parameter a using Equation (1).

(1) $$a=\sqrt{d^{2}*(h^{2}+k^{2}+l^{2})}$$

#### 2.2.5. Calorimetric Measurements

The heating power of the particles was determined using a calorimetric hyperthermia measurement setup. As a field generator a SINAC 12 SH, from EFD Induction GmbH, Freiburg im Braisgau, Germany, was used together with a FOTEMP 2 fiber optical temperature probe from Weidmann Technologies Deutschland GmbH, Dresden, Germany. The resolution of the FOTEMP 2 was 0.1 K with a standard resolution of ±0.3 K. Measurements were made for all synthesized samples and averaged for the three samples with the same x. Specific absorption rate (*SAR*) as a measure for the heating power of nanoparticles was determined in fluid and agarose samples. Fluid samples consisted of 0.5 mL deionized water with 1 wt% of particles. Agarose samples for immobilized particles contained 1 wt% particles in 0.5 mL of a 1 wt% agarose gel. The external alternating magnetic field was set to a frequency of 290 kHz and amplitudes of 40, 54 and 69 mT. For each particle sample, three fluid and three gel measurement samples were prepared, and the temperature increase was measured at each field amplitude with a sampling interval of 0.5 s. The temperature probe was placed in the center of the sample, and no sedimentation of the particles was observed within the measurement time. Second, *SAR* values were calculated using Equation (2) for each measurement. Last, the three *SAR* values for each particle and field amplitude were averaged. This procedure was used because *SAR* measurements are subject to a rather large statistical error that can be minimized by averaging.

(2) $$SAR=\frac{\Delta T}{\Delta t}*c*\frac{m_{S}}{m_{MNP}}$$

**Figure 1:**
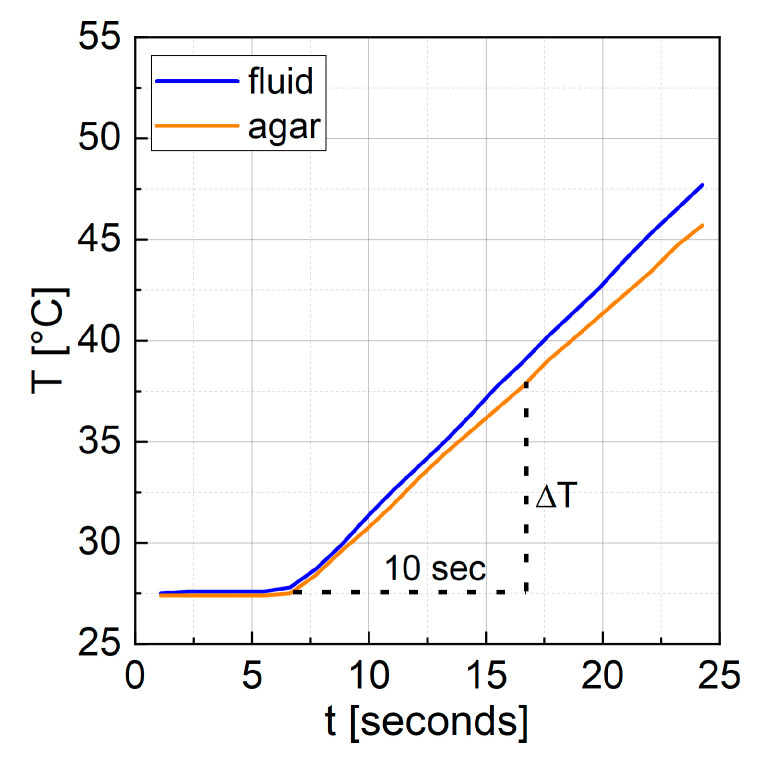
Representative temperature curve of a particle sample with cobalt content x = 0.3 measured as a fluid and in agar gel at 290 kHz and 69 mT. Δ*T* was extracted as an absolute value after 10 s of heating.

The *SAR* is given by Equation (2), where c is the heat capacity of water, $m_{S}$ is the mass of the sample and $m_{MNP}$ is the mass of particles within. The same heat capacity was used for the agarose samples, as the concentration of agarose in water of 1 wt% did not change the heat capacity significantly. Δt was set to 10 s and ΔT as an absolute value over this time span was extracted from the temperature curves (see Figure 1) by utilizing a MATLAB script for automatization. For further details on *SAR* measurements, refer to [30].

## 3. Results and Discussion

### 3.1. Series 1—Variation of Synthesis Parameters

#### 3.1.1. XRD

For each parameter set of series 1, a sample was characterized using XRD. The results can be seen in Table 2, where the crystallite size, the crystallinity and the lattice parameter a are given. Some samples did show additional crystalline phases in XRD and are marked red in Table 2. 

Most samples of series 1 were single phase CoFe_2_O_4_ and 7 samples were multi-phase CoFe_2_O_4_ plus NaCl and/or FeO(OH) with CoFe_2_O_4_ fractions between 35% and 89%. In one sample (O_2__85 °C_2 min_60 min_97 °C) Co(OH)_2_ and magnetite (Fe_3_O_4_) were indicated together with FeO(OH) and NaCl to form the most likely phase composition. Determining phase composition with XRD for our samples needs to be handled with care, as the diffractograms of magnetite, maghemite and cobalt ferrite in nanoparticulate form are difficult to distinguish. Quantification was carried out using the “direct derivation method” [31]. In Table 2 and Table 3, the samples with additional phases are marked red.

**Figure 2:**
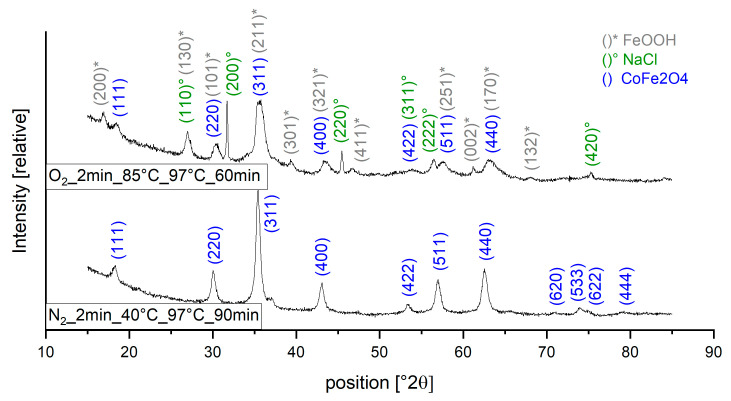
XRD spectra for sample O_2__2 min_85 °C_97 °C_60 min as an example for particles containing multiple phases and sample N_2__2 min_40 °C_97 °C_90 min as an example for pure cobalt ferrite particles.

Figure 2 shows typical diffraction patterns of our samples. The peaks of the nanocrystalline powders are rather low because of the large amounts of amorphous components, resulting in crystallinity values from 26.9 to 44.4%. The rise of the background between 15° and 30° 2θ is caused by air scattering. For the determination of the crystallinity, the diffraction pattern of a ZBH measurement must be subtracted beforehand, which reflects the influence of the air scattering. The amorphous components and thereby low reflection peaks complicate the phase identification: for reliable detection of a peak, an SNR > 3 is needed, a threshold that is not reached for some of the peaks in our XRD measurements. Phase identification for our samples therefore needs to be considered with care.

The crystallite sizes are between 10.3 and 18.0 nm determined with the “size-strain-analysis-R”-method (Rietveld) for the CoFe_2_O_4_ phase. The microstrain values are near zero ranging from 0% and 0.6% for single phase samples and between 0.6% and 1% for multi-phase samples.

Lattice parameters are derived from the interplanar spacing of the (311) plane, with former ranging between 8.342 Å and 8.426 Å, and most likely depend on the formation of a hydroxide phase. This may result in an iron deficit of the spinel phase, which is described in more detail below.

Only a partial correlation between magnetic properties (described in detail in the next chapter) or varied synthesis parameters and crystallite size can be found. For single varied parameters under N_2_ atmosphere, a correlation between synthesis parameter and crystal size can be seen—particles become larger with increasing d_react_ (15.5 to 16.4 nm), as well as with increasing d_add_ (11.4 to 17.4 nm) and increasing T_add_ (11.0 to 14.1 nm). Under O_2_ atmosphere, no correlation of size and synthesis parameters can be found. Therefore, other factors beyond the macroscopic particle size need to be considered when discussing the change in magnetic parameters with variations of the synthesis process. Crystallinity, determined from XRD measurements, also seems to correlate with few synthesis parameters. For both O_2_ and N_2_, increasing the end temperature of the reaction increases crystallinity (for N_2_ from 28.2 to 41.4%, for O_2_ from 30.7 to 43.6%), indicating a dependency of the formation of a well-structured crystal on the temperature to which the precipitate is heated.

#### 3.1.2. Magnetic Properties

**Figure 3:**
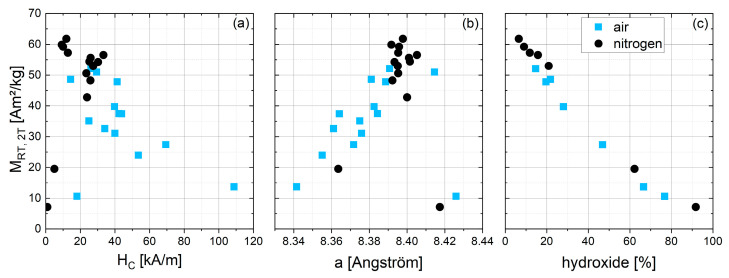
Correlation of M_RT,2T_ of series 1 samples with (**a**) coercivity H_C_, (**b**) lattice parameter a, and (**c**) estimated hydroxide content from Mössbauer spectra for selected samples of series 1.

**Figure 4:**
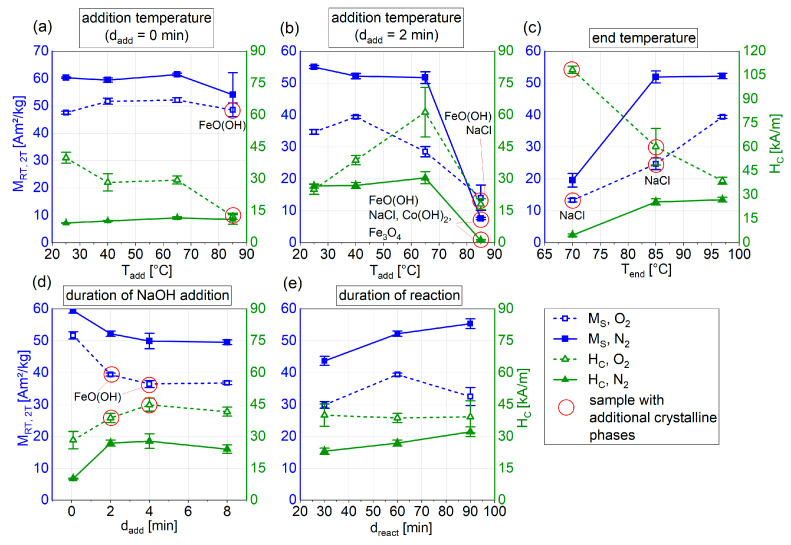
Mean H_C_ (green triangles) and M_RT,2T_ (blue squares), depending on the varied synthesis parameter (**a**) addition temperature with d_add_ 0 = min (**b**) addition temperature with d_add_ = 2 min (**c**) end temperature (**d**) duration of NaOH addition and (**e**) duration of reaction. Full symbols represent samples synthesized under the N_2_ atmosphere, while empty symbols represent the O_2_ atmosphere. Red circles mark samples with crystalline phases, apart from CoFe_2_O_4_, determined with XRD. Possible phases are indicated by the circles.

All samples of series 1 were characterized magnetically using VSM measurements at room temperature (RT). M_RT,2T_ and H_C_ were averaged for the three samples synthesized with the same parameter set. The results are listed in Table 3 and shown graphically in Figure 3 and Figure 4. 

Please note that the magnetization values at 2 T and 9 T for series 2 of this study, described below, were extracted from the measurement as high field magnetization values, while the M(H) curves indicate that the particles were not yet saturated at 9 T. Therefore, the measured values are named M_RT,2T_ and M_9T_ and not saturation magnetization M_S_.

All of the varied synthesis parameters show an influence on the resulting magnetic characteristics of the particles. Figure 3 gives an overview of the magnetization of all particles from series 1 at room temperature and a field of 2 T in dependence of H_C_, lattice parameter a and hydroxide content.

Concerning the used atmosphere, oxygen or nitrogen, an overall trend can be seen (Figure 3a): with values ranging from 12 to 108 kA/m and an average of 40.7 kA/m, the particles synthesized with O_2_ show significantly higher H_C_, compared with the reaction under N_2_, where H_C_ ranges from 1 to 32 kA/m with an average of 19.8 kA/m. M_RT,2T_, in contrast, is higher for N_2_ samples ranging from 7.6 to 61.5 Am^2^/kg with an average of 49.9 Am^2^/kg, and 13.3 to 52.1 Am^2^/kg with an average of 36.7 Am^2^/kg for O_2_ samples. Figure 3b shows the lattice parameter a, derived from the position of the (311) peak in the XRD data. A linear correlation can be seen between the lattice parameter and M_RT,2T_, whereby an increase in lattice parameter a correlates with a higher M_RT,2T_. A shift in lattice parameter of a crystal lattice can originate from a rearrangement of the ion distribution on the octahedral and tetrahedral sites [32]. However, since the effect on the magnetization is rather large, ion redistribution as the only reason for the increasing lattice parameter and decreasing magnetization seems unlikely. A more likely explanation is the formation of an additional phase of low magnetization—described in more detail below and as indicated by the Mössbauer spectra of some samples—pointing towards akageneite (FeO(OH)). Depending on pH during synthesis, akageneite can precipitate in an amorphous structure [33], explaining why XRD did find crystalline FeO(OH) only in some of these samples. The estimated amount of hydroxide material in those samples can be seen in Figure 3c and correlates well with the magnetization. The formation of such an iron-containing hydroxide phase would result in a slight excess of cobalt in the remaining particles, shifting the stoichiometry of CoFe_2_O_4_ towards more cobalt-rich phases such as FeCo_2_O_4_. Lattice parameters for CoFe_2_O_4_ are reported in the range of 8.371 Å [34] to 8.40 Å [35], while smaller lattice parameters have been observed for cobalt-rich ferrites. Ateia et al. [36] found the lattice parameter of Co_1.5_Fe_1.5_O_4_ to be 8.366 Å, while CoFe_2_O_4_ resulted in a = 8.382 Å in their study. Increasing Co content further to FeCo_2_O_4_, results in a lattice parameter a as small as 8.242 Å [37]. Discussing the formation of cobalt-rich phases, one needs to note that FeCo_2_O_4_ is stable only between 850 and 950 °C and decomposes into two spinel phases below 850 °C [38]. Therefore, we expect the cobalt-rich phase in our particles to have a stoichiometry close to Co_x_Fe_3−x_O_4_ with x ⪆ 1. Cedeno-Mattei et al. [20] and Whaba et al. [39] synthesized cobalt ferrites with varying x up to values larger than 1 and observed a slight decrease in lattice parameter a for x > 1 as well, supporting our assumption of decreasing lattice parameter with increasing cobalt content. Higher M_RT,2T_ and larger lattice parameters for particles synthesized under N_2_ indicate the suppression of the hydroxide phase under an inert atmosphere due to reduced oxidization. Looking at Figure 3a, an overall negative correlation between H_C_ and M_RT,2T_ can be seen—particles with a high H_C_ show low M_RT,2T_ and vice versa. This can be explained by the slightly higher H_C_ values found for x > 1 [40] and the lower net magnetization due to the antiferromagnetic character of the mainly amorphous hydroxide phase.

In the following section, the influence of the varied synthesis parameters will be discussed for each parameter. 

Looking at the varied temperatures during synthesis (addition temperature T_add_ and the end temperature T_end_ of the reaction, Figure 4a–c), thresholds seem to exist, which, if exceeded, lead to impurity phases beyond the CoFe_2_O_4_. Samples, where additional phases were observed via XRD, are marked red in Table 2 and Table 3 and Figure 4. 

The temperature T_add_ of the metal salt solution when NaOH is added for the precipitation process was evaluated for fast (d_add_ = 0 min) and slow (d_add_ = 2 min) addition of the alkaline medium. For a slow addition, particle M_RT,2T_ and H_C_ decrease drastically for T_add_ = 85 °C for both the O_2_ and N_2_ atmospheres (Figure 4b). This drop in the magnetic behavior could be related to the occurrence of crystalline nonmagnetic phases in the particles as detected by XRD measurements. Under N_2_ atmosphere, FeO(OH) and NaCl were detected in addition to CoFe_2_O_4_, while the sample synthesized under O_2_ is assumed not to contain any crystalline CoFe_2_O_4_. Instead, a small amount of Fe_3_O_4_, together with FeO(OH), NaCl and Co(OH), was indicated by XRD. Akageneite can precipitate in solutions containing Fe^3+^ ions, if pH < 4 or pH > 8 and a high concentration of chloride is present, which is the case for our synthesis method using iron- and cobalt chloride. Without chloride, goethite (α-FeO(OH)) would form under the same conditions [41]. This process starts around 55 °C and is enforced with rising temperature, [42] explaining the formation of akageneite that results from increased addition temperatures in our experiments. The precipitation of akageneite was also observed during the synthesis, as the metal salt solution turned opaque above a temperature of approximately 70 °C. 

For the slow addition of the alkaline medium and a T_add_ between 25 and 65 °C only O_2_ samples show an influence of T_add—_H_C_ increases with increasing T_add_ and M_RT,2T_ decreases for T_add_ = 65 °C. This indicates an influence of O_2_ on the hydroxylated ferric ions in the salt solution. Hydroxylation of ferric ions takes place at 1 < pH < 4, as does the rapid condensation of the hydroxylated ion–aquo complexes, to form ferrihydrite [41]. Co^2+^ ions, in contrast, need pH > 8 to hydroxylate and condensate [43]. A temperature increase of the metal salt solution before the alkaline medium is added, and while the metal salt solution is still strongly acidic, therefore most likely has an influence on the condensation of the ferrihydrites, matching the assumed formation of FeO(OH), either crystalline or amorphous. 

Compared with the slower addition of the alkaline medium over 2 min discussed above, increasing T_add_ when NaOH is added rapidly to the metal salt solution (d_add_ = 0 min, Figure 4a) results in different trends. For the N_2_ atmosphere, only a slight increase in H_C_ with increasing T_add_ and a slight drop in M_RT,2T_ for T_add_ = 85 °C can be seen. For the O_2_ atmosphere, T_add_ = 85 °C again leads to the detection of crystalline akageneite FeO(OH) in XRD measurements; however, most likely only very small amounts of akageneite are formed, as M_RT,2T_ does not decrease significantly. For T_add_ = 25–65 °C, H_C_ decreases with increasing T_add_. 

Looking at T_end_ (Figure 4c), which represents the maximum temperature that the reactants are heated up to after the precipitation, the O_2_ samples show remaining amounts of NaCl for T_end_ = 70 and 85 °C. For T_end_ = 70 °C, a very high H_C_ (108 kA/m) and a low M_RT,2T_ (20 Am^2^/kg) can be seen, indicating the incomplete formation of a CoFe_2_O_4_ crystal lattice. In XRD measurements of this sample, no other crystalline phase, apart from NaCl and CoFe_2_O_4_, was found; however, Mössbauer spectroscopy, as shown below does display a doublet subspectrum, indicating the presence of an antiferromagnetic phase, most likely an amorphous iron hydroxide, as discussed above. With increasing T_end_, H_C_ decreases and M_RT,2T_ increases, leading to particles with a high magnetization of almost 39.4 Am^2^/kg and a H_C_ of 38.7 kA/m for T_end_ = 97 °C. For synthesis under N_2_ atmosphere, a T_end_ of 70 °C seems also to be insufficient, leading to particles with a low M_RT,2T_ of 19.5 Am^2^/kg and no significant H_C_ and indicating that the formation of ferrimagnetic CoFe_2_O_4_ was hindered. T_end_ > 85 °C enables the formation of ferrimagnetic particles with M_RT,2T_ above 48 Am^2^/kg and H_C_ of 25 to 29 kA/m.

Apart from synthesis temperatures, the duration of NaOH addition and of the overall reaction were evaluated. Looking at the duration of NaOH addition (Figure 4d), all samples synthesized under N_2_ were pure CoFe_2_O_4_ derived from XRD and no crystalline impurities were found. For O_2_ atmosphere, samples with d_add_ = 2 and 4 min showed some crystalline FeO(OH) in XRD measurements. Interestingly, the sample synthesized with d_add_ = 8 min in O_2_ atmosphere did not show a second phase in XRD. Because all of the particles studied are only partially crystalline, and amorphous components contribute significantly, there are only few relatively low and broad reflections, making phase identification difficult. Therefore, it must be considered that the contribution of additional phases may be too small to allow reliable detection. Concerning magnetic properties, increasing d_add_ results in similar trends for O_2_ and N_2_ samples (Figure 4d). With increasing d_add_, leading to a slower increase in pH and thereby a slower precipitation process, H_C_ increases up to d_add_ = 4 min and slightly decreases again for d_add_ = 8 min. M_RT,2T_ simultaneously decreases with increasing d_add_, reaching a plateau around 50 Am^2^/kg for N_2_ samples and 36 Am^2^/kg for O_2_ samples. 

The duration of the reaction (d_react_), starting after NaOH addition and ending with the removal of the reactants from the water bath and the washing of the particles, has a minor influence on the resulting parameters (Figure 4e). For synthesis under N_2_ atmosphere, M_RT,2T_ can be increased by prolonging the reaction time with a maximum value of M_RT,2T_ = 55.3 Am^2^/kg for d_react_ = 90 min, while slightly increasing H_C_ at the same time. For O_2_ samples, H_C_ is not affected by the reaction time while M_RT, 2T_ has its maximum at a medium duration of reaction of 60 min. 

Summarizing the results of the first series of synthesis with a variation of synthesis parameters, we can conclude that, for high M_RT,2T_, the synthesis under nitrogen atmosphere should be favored, while synthesis under oxygen leads to higher H_C_. The increase in temperature of the salt solution when adding NaOH should not exceed 65 °C in any case, for synthesis under oxygen 40 °C may not be exceeded to prevent a decrease in M_RT,2T_. The end temperature of the reactants should be 97 °C, to prevent evaporation of the solvent by boiling while enabling crystallization and high M_RT,2T_. Duration of NaOH addition can be used to tune H_C_, but a trade-off between H_C_ and M_RT,2T_ needs to be made, wherefore 2 min is suggested as d_add_ for further experiments. The overall duration of the reaction should be 90 min for synthesis under N_2_ and 60 min for O_2_. The parameters for the synthesis of the samples in series 2 with varied amounts of cobalt were therefore chosen to be T_add_ = 40 °C, d_add_ = 2 min, T_end_ = 97 °C and d_react_ = 90 min.

#### 3.1.3. Mössbauer Spectroscopy

**Figure 5:**
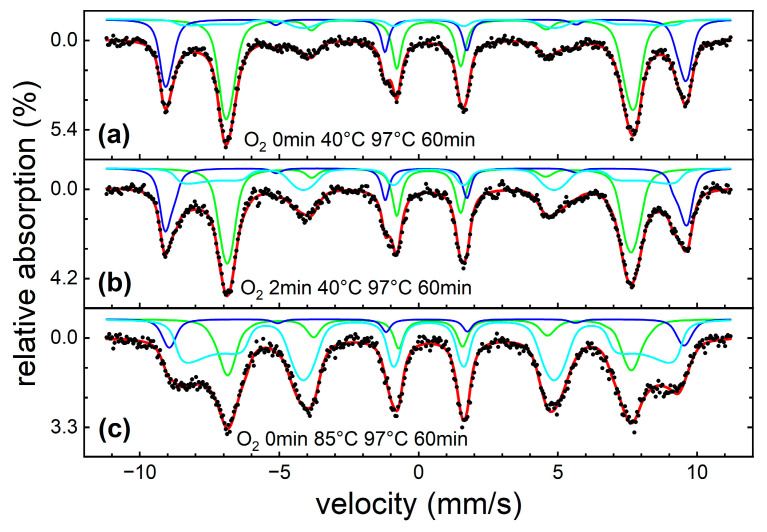
Mössbauer spectra of exemplary samples showing low (**a**), moderate (**b**), and high (**c**) hydroxide fraction recorded at 4 K in an external magnetic field of 8 T along the γ-ray propagation direction. Black dots represent experimental data points, red lines the overall theoretical fit function. Spinel subspectra corresponding to iron on A-sites (blue) and B-sites (green) were reproduced using narrow hyperfine field distributions, while the additional contribution assigned to an akageneite-like Fe^3+^ antiferromagnetic phase (cyan) was represented using a simulation of an ensemble of randomly oriented antiferromagnetic particles in an external magnetic field.

To better understand the magnetic structure as well as the composition of the particle powders prepared under different conditions, we performed an in-depth Mössbauer spectroscopy analysis. Spectra recorded at 4 K in an external magnetic field of 8 T parallel to the γ-ray propagation direction are shown in Figure 5, illustrating samples of very low (a), moderate (b), and high (c) hydroxide fraction appearing in addition to the expected spinel phase. For synthesis parameters as used in (a), the spectrum consists primarily of well-resolved contributions of Fe^3+^ in tetrahedral (A-site, blue) and octahedral (B-site, green) surroundings, as expected for CoFe_2_O_4_. Ideal synthesis parameters described above and used for the preparation of particle powders in series 2 with varying stoichiometry, as presented in the following section, resulted in hydroxide fractions far below 10%, wherefore this component will not be considered separately in the Mössbauer data analysis of series 2.

For higher hydroxide fractions, the antiferromagnetic character of this phase became more evident, resulting in the high intensity of lines 2 and 5, representing non-aligned spins [44] and characteristic lineshapes, respectively, as reported before for antiferromagnetic nanoparticles exposed to high magnetic fields [45]. This component was therefore reproduced by simulating an ensemble of randomly oriented antiferromagnetic particles in an external magnetic field, using literature values of exchange and anisotropy fields of bulk akageneite [46], closely matching the shape of the experimental spectra. Independent of XRD analysis, the identification of the byphase as akageneite can also be inferred from the temperature-dependent Mössbauer spectroscopy below. In general, the nanoparticles’ degree of spin frustration does not seem to exhibit a strong correlation to the amount of hydroxide, with the spinel phase showing moderate spin canting angles, based on observed subspectral line intensity ratios. As it is reasonable to assume that ferri- to antiferromagnetic interfaces of mixed spinel–hydroxide particles would result in strong interface spin frustration, this would indicate that spinel and hydroxide species grow as individual particles. 

**Figure 6:**
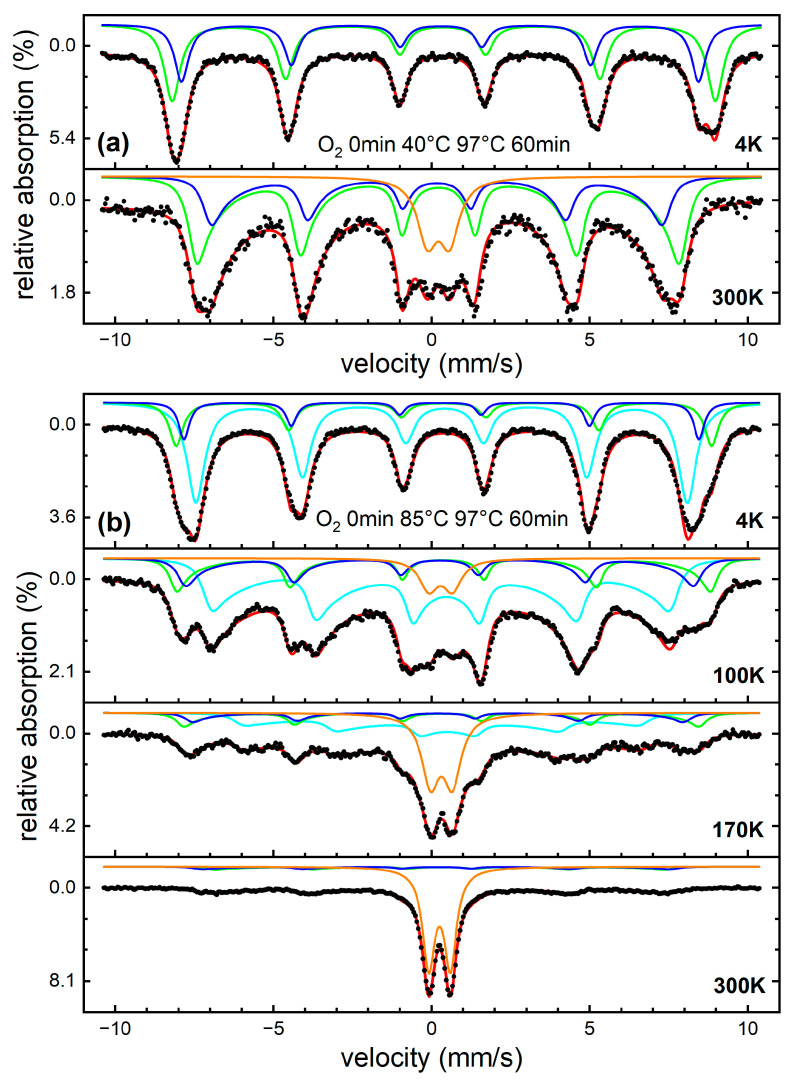
Mössbauer spectra recorded between 4 and 300 K for exemplary samples showing low (**a**) and high hydroxide contents (**b**), respectively. Black dots represent experimental data points, red lines the overall theoretical fit function. Asymmetric deformation was reproduced using a multilevel relaxation model within the slow relaxation regime [49]; spectra consist of A- and B-site spinel subspectra (blue and green), as well as akageneite in the antiferromagnetic low-temperature state (cyan). The high temperature doublet state (orange) is assigned to a superposition of superparamagnetic spinel particles and paramagnetic akageneite.

All nanoparticle powders were analyzed via Mössbauer spectroscopy between 4 and 300 K, to identify their composition and to examine them with regards to superparamagnetic relaxation effects. Figure 6a shows a sample composed almost entirely of spinel material. At elevated temperatures, a minor asymmetry of the absorption lines is visible, originating from beginning Néel-type superspin relaxation. A small doublet is also visible at this temperature, containing ca. 14% of spectral area. This could represent a fraction of the smallest spinel particles, already reaching fast superparamagnetic relaxation, but likely also contains small fractions of paramagnetic byphase, as becomes evident based on Figure 6b. At low temperatures, we see that the hydroxide subspectrum partially overlaps with spinel components due to similar hyperfine parameters: an isomer shift of ca. 0.48 mm/s (relative to α-Fe at RT), which is characteristic for Fe^3+^; a hyperfine magnetic field B_hf_ of ca. 48.2 T; and a nuclear quadrupole level shift 2ε of ca. −0.1 to −0.15 mm/s. These parameters match those reported previously for akageneite [47,48], in agreement with results from XRD analysis. This identification is further supported by studying the temperature-dependent decrease in B_hf_, yielding a Néel temperature of ca. 290 K, again in agreement with literature values of akageneite, before reaching the paramagnetic doublet state (orange) at room temperature. 

### 3.2. Series 2—Variation of Cobalt Fraction x

In a second series of experiments, Co_x_Fe_3−x_O_4_ samples with varying cobalt fraction x were synthesized with the same set of synthesis parameters under a nitrogen atmosphere. 

Table 4 gives an overview of the characterization of series 2 particles, including magnetometry and XRD-derived results. They will be described and discussed in detail in the following sections.

#### 3.2.1. TEM Analysis

**Figure 7:**
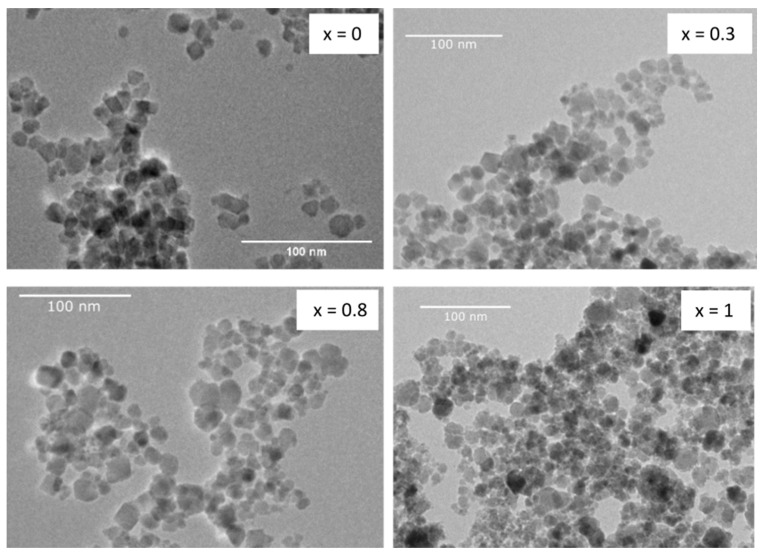
TEM micrographs of samples x = 0, 0.3, 0.8, 1—deposited from solutions of 5 mg/mL concentration.

TEM micrographs (Figure 7) show spherical to slightly irregular shaped particles that confirm the size range determined from XRD measurements between 12.4 and 16.6 nm. An influence of cobalt fraction x on the particle shape and size can be assumed: with increasing x, size distributions seem to become broader, with an increasing amount of very small particles, resulting in a rather inhomogeneous distribution for x = 1. Mean particle size determined by XRD did not show a clear trend with increasing x. One has to keep in mind that XRD only takes into account crystalline components, while the investigated particles most likely contain amorphous phases to some extent, leading to uncertainties regarding the size determination. 

#### 3.2.2. XRD

**Figure 8:**
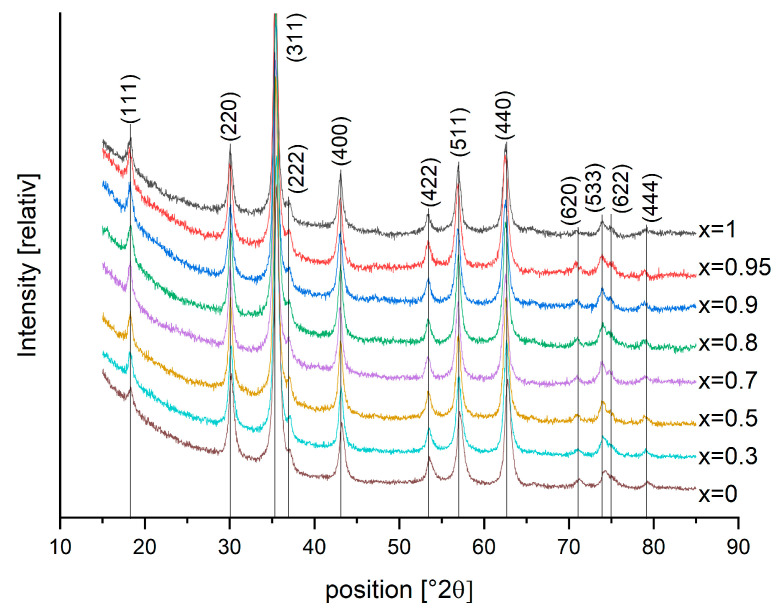
XRD diffractograms of all samples with varying Co fraction x. For better visibility, the curves are shifted on the *y* axis.

**Figure 9:**
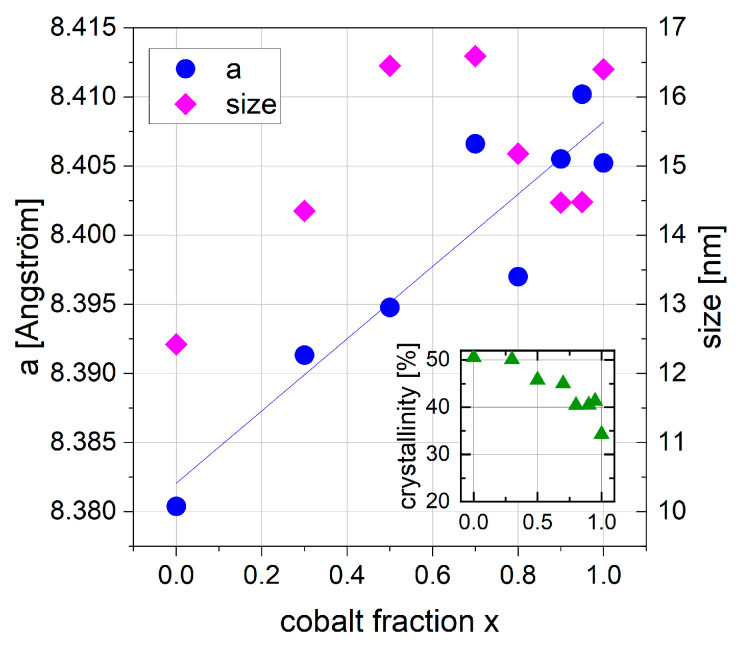
Lattice parameter a as blue dots (**left**) and crystallite size as pink rhombuses (**right**) plotted against cobalt fraction x. Inset shows crystallinity (green triangles) depending on cobalt fraction x.

Diffractograms of all series 2 samples can be seen in Figure 8. All samples were single-phase, identified with either a Fe_3_O_4_ (magnetite) reference card for x = 0 or CoFe_2_O_4_ reference card for x > 0. Co doping from magnetite to CoFe_2_O_4_ has an effect on the lattice spacing, but not on the crystal lattice structure, which remains cubic. The lattice spacing d and thus the lattice parameter a become about 0.4% larger, which is shown in Figure 9. The crystallite size for the series 2 samples varies from 12.4 nm (x = 0) up to 16.6 nm (x = 0.7), the micro strain from 0% (x = 0) up to 0.13% (x = 0.7), determined with the “size-strain-analysis-R”-method (Rietveld) for the CoFe_2_O_4_ phase.

The Co doping of magnetite up to cobalt ferrite increases the calculated lattice parameter from 8.38 Å to 8.41 Å (Figure 9). Thus, our CoFe_2_O_4_ sample has a slightly larger lattice parameter compared with reference card values from the ICDD, ICSD or COD database, where the lattice parameter for CoFe_2_O_4_ varies from 8.350 Å up to 8.400 Å. An increase in lattice parameter a with increasing cobalt fraction can be found in the literature; Kumar et al. 2021 [23] found a to increase from 8.351 to 8.395 Å with x increasing from 0 to 0.8. In contrast, the opposite trend is also described, with a decreasing lattice parameter with increasing x [27]. The mechanisms behind these contrasting phenomena seem unclear and to be influenced by several factors. Oxidation of magnetite to maghemite for particles with low cobalt content could be one factor, leading to smaller lattice parameters for lower x. The crystallinity varies from 40.4% up to 50.6%, with a decreasing trend for increasing x (see Figure 9, inset), indicating higher amounts of amorphous components for higher cobalt doping. Mössbauer spectroscopy did not find significant amounts of additional iron containing amorphous phases in series 2 samples, which indicates the presence of some cobalt ferrite in an amorphous structure.

#### 3.2.3. Magnetic Properties

**Figure 10:**
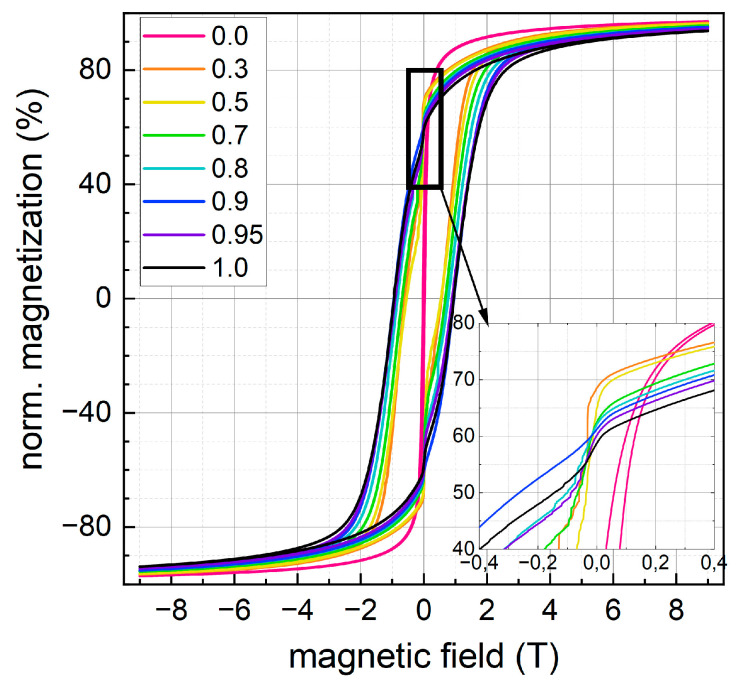
M(H) loops recorded at 4.3 K up to maximum fields of 9 T for x = 0.0–1.0. M(H) data are normalized to the saturation magnetization Ms, estimated by extrapolation of the high-field region.

**Figure 11:**
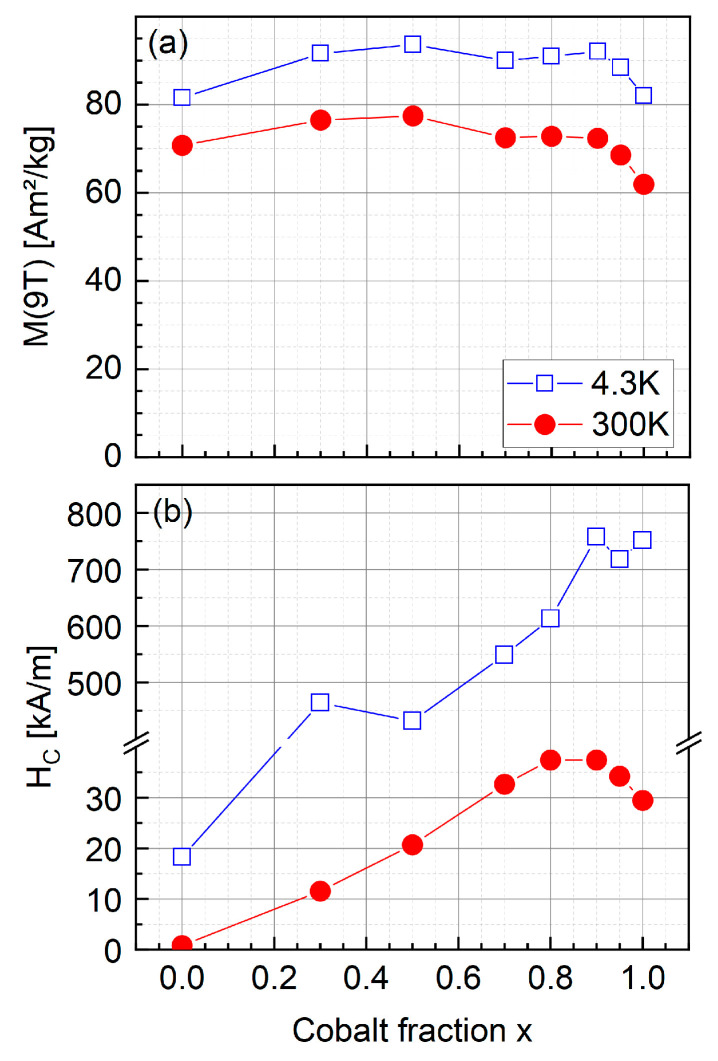
High-field magnetization M_9T_ (**a**) and coercivity H_C_ (**b**) observed at 4.3 K (open symbols) and 300 K (full symbols), respectively.

To evaluate the magnetic parameters strongly varying upon the change in Co-content x, M(H) hysteresis loops were recorded at 4.3 K up to 9 T as displayed in Figure 10, as well as at 300 K. All samples, except for the x = 0.0 sample, show open hysteresis loops with relatively high coercive fields in the 1 T (ca. 800 kA/m) range, typical for cobalt ferrite nanoparticles, and high-field magnetization values close to 90 Am^2^/kg. A clearly visible decrease in magnetization when going through zero magnetic field indicates the presence of a soft magnetic fraction, showing a relevant contribution for low Co-content and representing a small fraction of particles of lower magnetic anisotropy. Studying the cobalt fraction x-dependent trend in the 9 T magnetization M_9T_ at 4.3 K and 300 K (see Figure 11), as extracted from the data in Figure 10, we observe a maximum at ca. x = 0.5. Lower values of M_9T_ for higher x could be explained by slightly higher degrees of spin frustration due to higher magnetic anisotropy in Co-rich spinel material, as discussed in more detail in the Mössbauer spectroscopy section. At the same time, the decrease in magnetization when going towards x = 0.0 can be assigned to oxidation, reducing the Fe^2+^ fraction in the spinel particles and thereby their saturation magnetization when vacancies replace B-site Fe^2+^, reducing the B-site sublattice magnetization. One has to keep in mind that a magnetite/maghemite transformation does not necessarily require aerial oxidation and can also happen under inert conditions by desorption of Fe^2+^ ions and hydroxylation in the solvent from the surface of particles, leaving behind a cationic vacancy and only trivalent Fe ions. As a result, oxide layers of ca. 2–3 nm may form within days to weeks, composed chiefly of maghemite, which explains the decrease of the overall magnetization of these oxidized samples [41,50]. At the same time, the difference between the M_9T_ values at 300 K and 4.3 K becomes smaller when considering lower Co-fractions, which could be attributed to the higher Curie temperature of magnetite and maghemite, as compared to cobalt ferrite with T_C_ ≈ 790 K [51].

A monotonous increase in coercivity H_C_ can be observed at 4.3 K when moving from low to high Co-fractions, with a maximum at ca. 750 kA/m for x = 0.9–1.0, while a maximum of ca. 35–40 kA/m is visible at x = 0.8–0.9 at 300 K, followed by a slight decrease when reaching even higher cobalt fractions. A maximum for H_C_ with medium cobalt ratios and a decrease of H_C_ when cobalt ratio x approaches 1 can be found in the literature: Yu et al. [21] found a maximum of H_C_ for x = 0.6 and stated that the co-existence of Fe^2+^ and Co^2+^ ions seems to enhance anisotropy. Kumar et al. [23] found H_C_ to be highest for x = 0.4–0.6 and suggested that with higher x the particle structure changed from single to multidomain, indicated by the increasing particle size with increasing x. A slightly increasing trend for particle size with increasing cobalt content can also be assumed for our particles (see Figure 9).

#### 3.2.4. Mössbauer Spectroscopy

**Figure 12:**
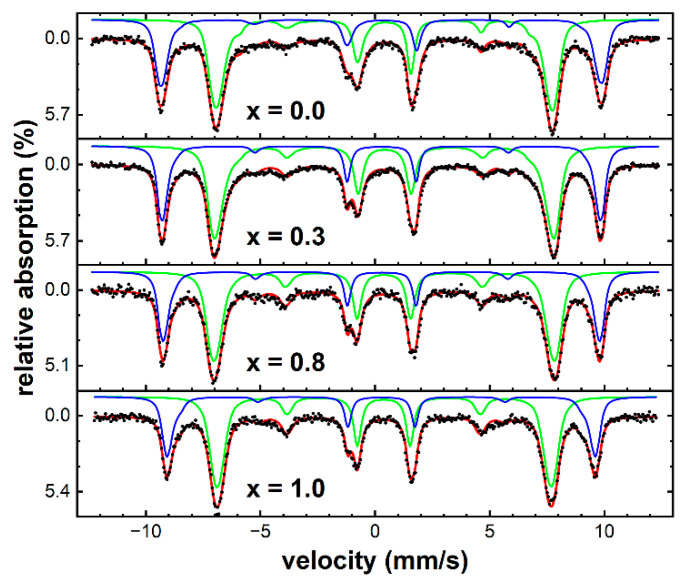
Mössbauer spectra of exemplary samples with cobalt content x = 0.0, 0.3, 0.8 and 1.0 recorded at 4 K in an external magnetic field of 8 T along the γ-ray propagation direction. Black dots represent experimental data points, red lines the overall theoretical fit function. Spinel subspectra corresponding to iron on A-sites (blue) and B-sites (green) were reproduced using narrow hyperfine field distributions.

Mössbauer spectra of spinel particles with x = 0.0 to 1.0 recorded at 4 K in a magnetic field of 8 T (Figure 12) do not exhibit the characteristic features assigned to hydroxides, as discussed above for series 1. As a result, this fraction was disregarded in further analysis, showing subspectra of octahedral (green) and tetrahedral (blue) Fe sites. However, a slight inner shoulder is visible for the B-site sextet subspectrum for x = 0.0 and x = 0.3, which could originate from a minor Fe^2+^ fraction. It seems reasonable to assume that for x = 0.0 the particles consist mainly of maghemite with a small magnetite core, limited to less than 20% of the particle volume based on the fine structure of the B-site sextet. Comparing relative spectral areas of A- and B-site subspectra, we find a ratio R_AB_ close to 0.6, as expected for maghemite, while pure magnetite would translate to R_AB_ = 0.5. Upon rising cobalt content, R_AB_ decreases monotonously until reaching a value of ca. 0.52 for x = 1.0, corresponding to an inversion parameter of ca. 0.68, which represents a mainly random ion placement with only a very minor B-site preference of Co^2+^ ions.

Although the degree of spin frustration is moderate in all samples, as indicated by the low intensity of lines 2 and 5, average spin canting angles show a slight increase from ca. 19° (x = 0.0) up to ca. 24° (x = 1.0). This could be explained by the higher magnetocrystalline anisotropy of cobalt ferrite as compared with iron oxides, which could add to the slight decrease in M_9T_ from x = 0.5 to x = 1.0 observed in magnetometry experiments. Considering the average spin misalignment angle θ_(8T)_ of ca. 20° for x = 0.5, determined in an external field of 8 T at 4 K, a saturation magnetization M_S_ of ca. 99.3 Am^2^/kg can be estimated via M_(8T)_ ≈ M_S_ · cos(θ_(8T)_) from the corresponding magnetization M_(8T)_ ≈ 93.3 Am^2^/kg. This value is far higher than expected when assuming an inverse spinel structure, as often discussed for bulk cobalt ferrite; however, it can be explained in terms of the mainly random placement of Co^2+^ ions [52], further supporting findings on inversion parameters discussed above.

#### 3.2.5. *SAR*

**Figure 13:**
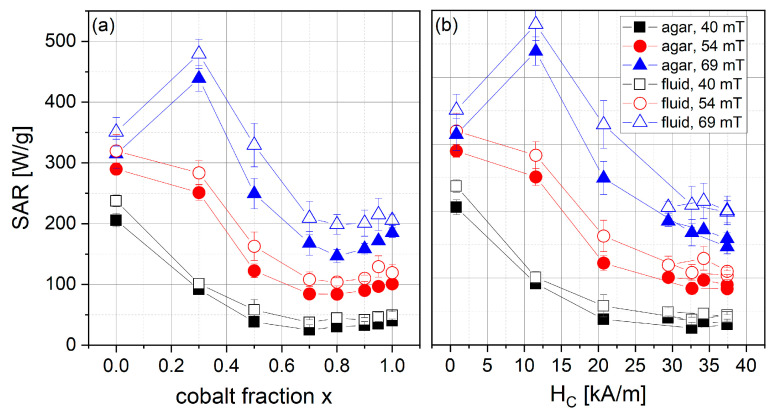
Mean *SAR* values and standard deviation for samples of series 2 in agar gel or fluid samples at varying field amplitude and a frequency of 290 kHz depending on (**a**) cobalt fraction x or (**b**) coercivity.

*SAR* was determined using a calorimetric measurement setup for particles in fluid and immobilized in agar gel at three different field amplitudes. The results can be seen in Figure 13. In general, *SAR* values measured in fluids are slightly higher than those measured in agar gel for the same sample. This indicates that the mechanism of heat generation can be attributed partially to a physical rotation of the particle that is prohibited in agar gel. A physical rotation of the particle can be expected for our particles, since their anisotropy constant is rather high, as can be seen from the high coercivity values. High magnetic anisotropy leads to a blocking of the intrinsic rotation of the magnetic moment within a particle and thus a physical rotation of the particle to follow the external magnetic field. However, the differences in *SAR* for fluid and agar samples are small, indicating that the particles may not have been completely immobilized in the agar gel. The influence of the used excitation field amplitude is as expected: with higher external field amplitudes, *SAR* values increase, leading to highest *SAR* values for the highest field of 69 mT. *SAR* also depends on the cobalt fraction x, with a maximum of 480 W/g (fluid sample, 69 mT) for x = 0.3 and a decrease in *SAR* for higher cobalt fractions. For x > 0.8, a slight increase in *SAR* can be seen, which might be related to the decreasing H_C_ for those samples (see Figure 11). Looking at Figure 13b, one can see that *SAR* monotonously decreases with increasing H_C_, after a maximum for H_C_ = 11.54 kA/m. For higher H_C_, the particles seem to be too magnetically hard to be heated efficiently by the used external field. The external field amplitude needs to be two or three times the coercivity as a rough guideline; however, depending on the particle size distribution and the anisotropy field distribution, even larger field amplitudes may be required to switch the particles’ magnetization [53]. In our case, the magnetization of particles with H_C_ higher than 12 kA/m, about a fourth of the external field amplitude, no longer switch efficiently, leading to the conclusion that the limiting factor for the heating power of the particles is the available external field amplitude. With the development of field generators producing higher field amplitudes for extracorporeal magnetic heating applications, this limitation can be overcome and the full potential of hard magnetic particles can be exploited.

## 4. Conclusions

We synthesized cobalt ferrite nanoparticles in the size range of 10 to 18 nm using a wet co-precipitation method. By varying the synthesis parameters in the first series of experiments over a broad range, we were able to identify thresholds that need to be met for the synthesis of crystalline and of pure cobalt ferrite nanoparticles and to prevent the formation of additional phases. Akageneite was found in some samples, indicated by XRD and Mössbauer spectroscopy, leading to a decrease in magnetization. Depending on the synthesis parameters, coercivities of up to 60 kA/m at room temperature and magnetizations (room temperature, 2 T) of 60 Am^2^/kg can be achieved. Second, the substitution of Fe^2+^ with Co^2+^ in Co_x_Fe_3−x_O_4_ with 0 < x < 1 leads to a linear increase in H_C_ up to x = 0.8, enabling the application-specific tuning of the particles’ magnetic behavior from a soft to a hard magnetism. For x > 0.8, H_C_ slightly decreases. High magnetization values for all Co_x_Fe_3−x_O_4_ particles, regardless of their cobalt fraction x, between 62 to 77 Am^2^/kg (room temperature, 9 T), were measured as a result of the optimized synthesis parameters found in the first part of this study. In-field Mössbauer spectroscopy experiments revealed moderate spin canting angles and a mainly random ion placement, both attributing to the observed high magnetization values. *SAR* values of up to 480 W/g (290 kHz, 69 mT) were measured for particles with x = 0.3, leading to the conclusion that the available external magnetic field amplitude is the limiting factor for the efficient heating of the hard magnetic high coercivity samples with higher x. The herein presented particles are promising candidates for extracorporeal magnetic heating applications with high magnetic field amplitudes.

## Figures and Tables

**Table 1:** Varied synthesis parameters and used values. Bold type values are declared as standard and used during the variation of other parameters.

Parameter	Abbreviation	Unit	Used Values			
Duration of NaOH addition	d_add_	min	0	**2**	4	8
Addition temperature	T_add_	°C	25	**40**	65	88
End temperature	T_end_	°C	70	85	**97**	
Duration of reaction	d_react_	min	30	**60**	90	

**Table 2:** Size, crystallinity and lattice parameter a for all parameter sets. The varied parameters are written in bold. Red values represent samples with additional crystalline phases, apart from CoFe_2_O_4_, determined by XRD.

				O_2_ Atmosphere			N_2_ Atmosphere		
d_add_	T_add_	T_end_	d_react_	Size	Crystallinity	a	Size	Crystallinity	a
min	°C	°C	min	nm	%	Å	nm	%	Å
**0**	40	97	60	12.9	42.7	8.391	11.4	43.6	8.396
**2**				12.6	43.6	8.383	15.7	41.4	8.395
**4**				12.0	35.6	8.364	17.0	37.8	8.392
**8**				13.6	40.0	8.384	17.4	36.3	8.395
0	**25**	97	60	13.3	42.3	8.389	11.0	36.0	8.392
	**40**			12.9	42.7	8.391	11.4	43.6	8.396
	**65**			13.8	26.9	8.415	12.1	44.4	8.398
	**85**			13.8	38.9	8.381	14.1	38.4	8.395
2	**25**	97	60	11.3	33.0	8.375	14.4	38.5	8.401
	**40**			12.6	43.6	8.383	15.7	41.4	8.395
	**65**			13.0	33.3	8.372	18.0	36.9	8.393
	**85**			14.2	32.7	8.426	12.5	28.5	8.417
2	40	**70**	60	12.8	30.7	8.342	10.3	28.2	8.364
		**85**		11.9	35.4	8.355	15.4	38.4	8.402
		**97**		12.6	43.6	8.383	15.7	41.4	8.395
2	40	97	**30**	12.0	34.8	8.376	15.5	33.1	8.400
			**60**	12.6	43.6	8.383	15.7	41.4	8.395
			**90**	11.9	37.4	8.361	16.4	34.3	8.405

**Table 3:** Mean H_C_ and M_RT,2T_ with standard deviation for all parameter sets. The varied parameters are written in bold. Red values represent samples with additional crystalline phases, apart from CoFe_2_O_4_, determined by XRD.

				O_2_ Atmosphere		N_2_ Atmosphere	
d_add_	T_add_	T_end_	d_react_	M_RT,2T_	H_C_	M_RT,2T_	H_C_
[min]	[°C]	[°C]	[min]	[Am^2^/kg]	[kA/m]	[Am^2^/kg]	[kA/m]
**0**	40	97	60	51.67 ± 1.1	28.30 ± 4.1	59.44 ± 0.8	10.16 ± 0.2
**2**				39.40 ± 0.5	38.72 ± 2.2	52.21 ± 0.8	26.87 ± 1.4
**4**				36.44 ± 1.1	44.91 ± 3.0	49.90 ± 2.4	27.81 ± 3.4
**8**				36.79 ± 0.5	41.65 ± 2.1	49.54 ± 0.8	24.05 ± 2.0
0	**25**	97	60	47.53 ± 0.5	39.84 ± 2.6	60.27 ± 0.3	9.22 ± 0.1
	**40**			51.67 ± 1.1	28.30 ± 4.1	59.44 ± 0.8	10.16 ± 0.2
	**65**			52.13 ± 0.8	29.41 ± 1.8	61.47 ± 0.5	11.63 ± 0.5
	**85**			48.51 ± 2.5	12.33 ± 1.5	54.08 ± 8.0	10.86 ± 2.3
2	**25**	97	60	34.70 ± 0.8	24.98 ± 2.3	55.03 ± 0.5	26.60 ± 0.9
	**40**			39.40 ± 0.5	38.72 ± 2.2	52.21 ± 0.8	26.87 ± 1.4
	**65**			28.49 ± 1.6	61.28 ± 11.6	51.74 ± 1.8	30.45 ± 2.8
	**85**			14.06 ± 4.0	18.11 ± 2.6	7.55 ± 0.4	1.01 ± 0.4
2	40	**70**	60	13.27 ± 0.6	108.31 ± 2.3	19.52 ± 2.1	4.65 ± 0.7
		**85**		24.70 ± 1.8	60.14 ± 11.4	51.89 ± 1.9	25.50 ± 1.9
		**97**		39.40 ± 0.5	38.72 ± 2.2	52.21 ± 0.8	26.87 ± 1.4
2	40	97	**30**	29.90 ± 1.0	40.00 ± 5.3	43.68 ± 1.4	23.05 ± 1.4
			**60**	39.40 ± 0.5	38.72 ± 2.2	52.21 ± 0.8	26.87 ± 1.4
			**90**	32.51 ± 2.8	39.19 ± 7.6	55.33 ± 1.5	32.22 ± 2.3

**Table 4:** Results of magnetometry and XRD for samples of series 2 with varied cobalt fraction x. H_C_ and M_9T_ were measured at 5 and 300 K with a maximum field of 9 T.

x	Co^2+^/ Fe^2+^+Fe^3+^	H_C_ @ 5 K [kA/m]	H_C_ @ 300 K [kA/m]	M_9T_ @ 5 K [Am^2^/kg]	M_9T_ @ 300 K [Am^2^/kg]	a [Å]	Size [nm]	Crystallinity [%]
0	0.00	18.31	0.80	81.57	70.77	8.380	12.4	50.6
0.3	0.11	464.07	11.54	91.62	76.52	8.391	14.4	50.1
0.5	0.20	431.43	20.70	93.65	77.43	8.395	16.5	45.8
0.7	0.30	548.44	32.64	90.01	72.48	8.407	16.6	45.0
0.8	0.36	612.92	37.41	90.97	72.81	8.397	15.2	40.4
0.9	0.43	757.79	37.41	92.08	72.38	8.406	14.5	40.5
0.95	0.46	717.99	34.23	88.41	68.57	8.410	14.5	41.3
1	0.50	751.42	29.45	81.98	61.92	8.405	16.4	34.3

## Data Availability

Data are available on reasonable request from the corresponding author.
